# Influence of Implant Neck Design on Peri-Implant Tissue Dimensions: A Comparative Study in Dogs

**DOI:** 10.3390/ma11102007

**Published:** 2018-10-17

**Authors:** José Luis Calvo-Guirado, Raúl Jiménez-Soto, Carlos Pérez Albacete-Martínez, Manuel Fernández-Domínguez, Sérgio Alexandre Gehrke, José Eduardo Maté-Sánchez de Val

**Affiliations:** 1 Faculty of Health Sciences, Universidad Católica San Antonio de Murcia, Murcia 30107, Spain; drjsoto@yahoo.com (R.J.-S.); cperezalbacete@ucam.edu (C.P.A.-M.); jemate@ucam.edu (J.E.M.-S.d.V.); 2 Faculty of Dentistry, Department of Oral and Implant Dentistry, CEU San Pablo University, Madrid Hospital Group, Madrid 28040, Spain; clinferfun@yahoo.es; 3 Biotecnos Research Center, Montevideo 11100, Uruguay; sergio.gehrke@hotmail.com

**Keywords:** bone levels, dental implants, neck design, soft tissue dimensions

## Abstract

This in vivo study assessed (hard and soft) peri-implant tissue remodeling around implants with micro-ring and open-thread neck designs placed in a dog model. Twenty histological sections corresponding to four different implant designs that were placed in America Foxhound dogs were obtained from previous studies. All the implants had been placed under identical conditions and were divided into four groups: Group A, micro-rings on implant neck plus 0.5 mm refined surface; Group B, micro-rings on implant neck; Group C, open-thread neck; and, Group D, double-spiral neck. Eight weeks after surgery, the integrated implants were removed and processed for histological examination. Crestal bone loss and bone-to-implant contact was greater for micro-ring necks than open-thread necks. Soft tissues showed significant differences on both buccal and lingual aspects, so that the distance from peri-implant mucosa to the apical portion of the barrier epithelium was smaller in the micro-ring groups. So, in spite of generating greater bone-to-implant contact, implants with micro rings produced more bone loss than open-thread implants. Moreover, the outcomes that were obtained IPX implants smooth neck design produced less bone loss in the cervical area, following by Facility implants when compared with the other open thread and microthreaded implant designs. Implant thread design can influence on bone remodeling in the cervical area, related to bundle bone preservation.

## 1. Introduction

The long-term success and predictability of implant-supported restorations depend on maintaining peri-implant hard and soft tissues [1,2,3]. During the first year of function, bone resorption will be of 1.5 to 2 mm, generally considered as a normal physiologic process [1]. Thereafter, an annual bone loss of 0.2 mm can be expected under normal circumstances [4,5]. The implant’s neck design may reduce marginal bone loss [6,7], and many different implant designs have attempted to preserve bone height after implant installation [8]. The implant neck design aims to reduce stress on the bone surrounding the implant and to stimulate the bone for remodeling.

It has been observed that the introduction of micro-rings on the implant neck may reduce early bone loss [9,10], and some authors suggest that micro-rings have the effect of limiting marginal bone loss in the presence of loading forces [11], the load transfer characteristics of the implant being dependent on the size and design of the implant neck [12,13]. In fact, the optimal load distribution offered by the micro-ring feature counteracts marginal bone loss [9,14]; it also enhances bone-to-implant contact. 

Calvo-Guirado et al. reported limited implant crestal bone loss (0.90 mm ± 0.26 mm) and a 100% implant survival rate after a 5-year follow-up with immediately restored implants with a neck with rough surface and micro-threads, placed in the anterior maxillary/esthetic zone and immediately restored with non-occlusal loading [15]. The same authors measured soft tissue thickness and marginal bone loss around dental implants with sloped (30°) micro-threaded shoulders as compared with conventional micro-threaded straight design implants that were placed in immediate post-extraction sites with immediate loading in an animal model. Both types of implants generated similar soft tissue thickness and marginal bone loss after a three-month follow-up [16].

Surface characteristics also have a significant influence on marginal bone loss. In the case of hybrid implants with micro-rings and flat surfaces, most of the implants present alveolar bone loss over the entire length of the flat surface, as far as the first thread [17], because the un-roughened surface of the implants fails to distribute occlusal loads adequately [18]. But implants with micro-rings and textured surfaces allow tissue ingrowth [19,20]. The surface microstructure varies depending on the implant surface treatment, which can modify stress distribution, cell response to the implant surface, and implant osseointegration. A systematic review by Smeerts et al. described five different implant surfaces found to promote recruitment, adhesion, and proliferation of osteogenic as well as fibroblastic cells, all achieving a high degree of hard and soft tissue integration and high levels of bone-to-implant contact [21].

Another study of 47 implants with micro-rings on the neck reported that bone loss around the implants was not significant after a two-year follow-up [18]. Calvo-Guirado et al. found minimal marginal bone loss and a 100% implant survival rate over the 3-year follow-up for immediate implants with micro-ring necks subjected to immediate non-occlusal loading [22]. In another study, implants with a polished neck of 0.8 mm plus one micro-ring and a roughened area of 2 mm was found to reduce buccal bone resorption [23].

In addition to implants that were designed with micro-rings on the neck, another neck design presents an open-thread, achieved by prolonging the spiral of the implant body over the neck. It has been suggested that this implant neck design shows better characteristics for load distribution, counteracting marginal bone resorption [24,25]. Preclinical studies have shown that, when compared with flat necks, the open-thread design increases bone-to-implant contact [26,27], providing greater preservation of crestal bone height [28,29].

To date, no consensus has been reached as to the effectiveness and influence of implant macro- and micro-design on marginal bone loss. The aim of this study was to evaluate the influence of implant neck design on soft and hard tissue remodeling around implants placed at crestal level, with abutment loading at the time of placement; all of the implant systems assayed had conical implant-abutment connections to reduce the shear stresses at the bone-to-implant connection [9,27].

## 2. Materials and Methods

The samples that were used in this study were obtained from previous studies that also assayed the four different implant systems selected for this trial. Inclusion criteria were as follows: implants placed crestally in fresh extraction sockets of dog mandibles, with platform switching, all carried out applying the same animal protocol, surgical protocol, healing period, and sample preparation. All of the samples received an abutment at the time of implant placement.

### 2.1. Animal Protocol

All samples that were used in the present study were obtained from previous studies performed using an American foxhound animal model. The animals were aged approximately one year and weighed 14–15 kg. All the earlier studies were approved by the Ethics Committee for Animal Research, ensuring that each study protocol fulfilled guidelines that were established by the European Union Council Directive 2010/63/UE. The project number of the four different experiments was A1320141102 (Murcia Agriculture and Water Ministry, Murcia, Spain).

The animals were fed a daily pellet diet. All animals presented intact maxillas, without any general occlusal trauma or oral viral or fungal lesions. Clinical examination determined that the dogs were in good general health, with no systemic involvement.

### 2.2. Sample Selection

**Figure 1:**
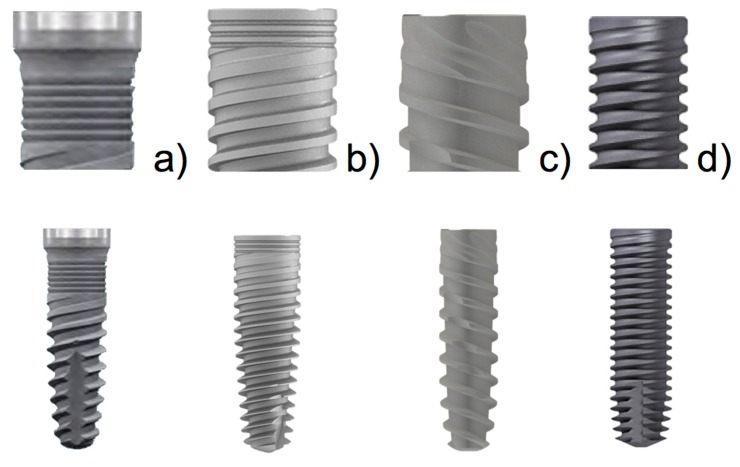
Implants used in the experiment: (**a**) Blue Sky, Bredent^®^; (**b**) C1, MIS^®^; (**c**) IPX, Galimplant^®^; and (**d**) Facility, Neodent^®^.

Five slides of four dental implant models with different designs were selected according to their macro- and micro-characteristics: Group A: Blue Sky implant (Bredent medical GMBH & Co. KG, Senden, Germany), 3.5 mm diameter and 10 mm length with a micro-ring neck plus a 0.5 mm refined surface; Group B: MIS C1 implant (MIS Implants Technologies Ltd, Tel Aviv, Israel), 4 mm diameter and 10 mm length, with a micro-ring neck; Group C: IPX implant (Galimplant, Sarria, Lugo, Spain), 4 mm diameter and 10 mm length, with an open-thread neck; Group D: Facility implant (Neodent, Instradent AG, Basel, Switzerland), 2.9 mm diameter and 10 mm length, with an (open thread) double-spiral on neck. Figure 1 illustrates the four implant designs.

### 2.3. Histological Preparation

**Figure 2:**
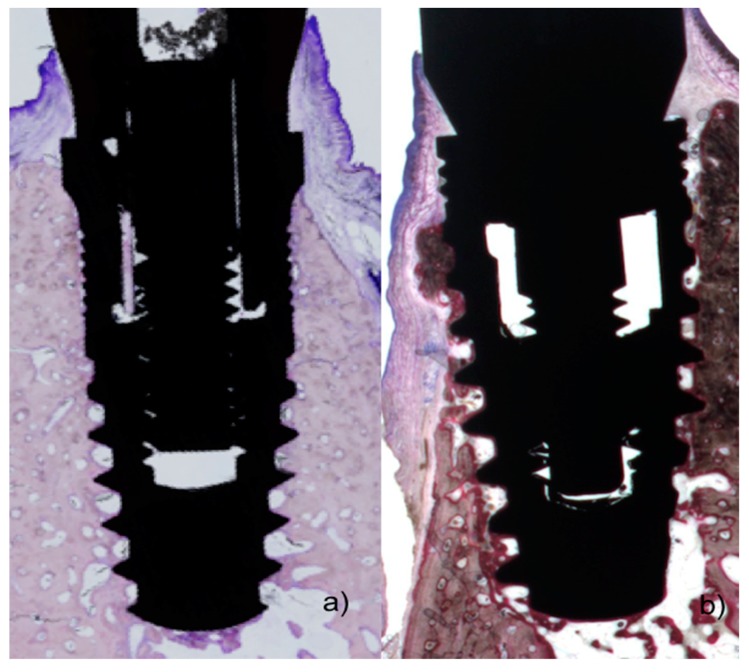
(**a**) Histological preparations representing the outcome after 8 weeks healing showing polished neck and micro-ring neck design of the Blue Sky implant; and (**b**) outcome after eight weeks healing of micro-ring neck design of the C1 implant.

**Figure 3:**
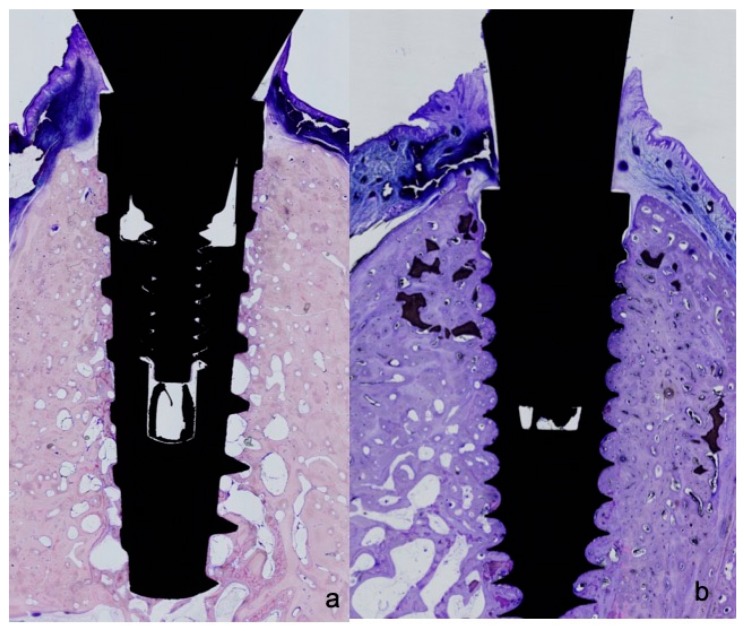
(**a**) Histological preparations representing the outcome after 8 weeks healing showing open-thread neck design of the IPX implant; and, (**b**) after eight weeks healing showing (open-thread) double-spiral neck design of the Facility implant.

All biopsies were processed using the same protocol, performing ground sectioning according to the method that was described by Donath & Breuner (1982) [27], designed to evaluate peri-implant soft tissue healing and bone remodeling. Samples were dehydrated in increasing grades of ethanol up to 100% and were embedded in a glycol methacrylate resin (Technovit 7200 VLC, Kulzer, Wehrheim, Germany). Then, the samples were polymerized and sectioned at the buccal-lingual plane using a diamond saw (Exakt, Apparatebau, Norderstedt, Germany). Sections were cut from each biopsy unit, from the center of the implant using a high-precision diamond disk to about 100 μm thickness and ground to approximately 40 μm final thickness with an Exakt 400s CS grinding device (Exakt, Apparatebau, Norderstedt, Germany). Each section was stained using toluidine blue stain (Figure 2 and Figure 3).

### 2.4. Histometric Evaluation

**Figure 4:**
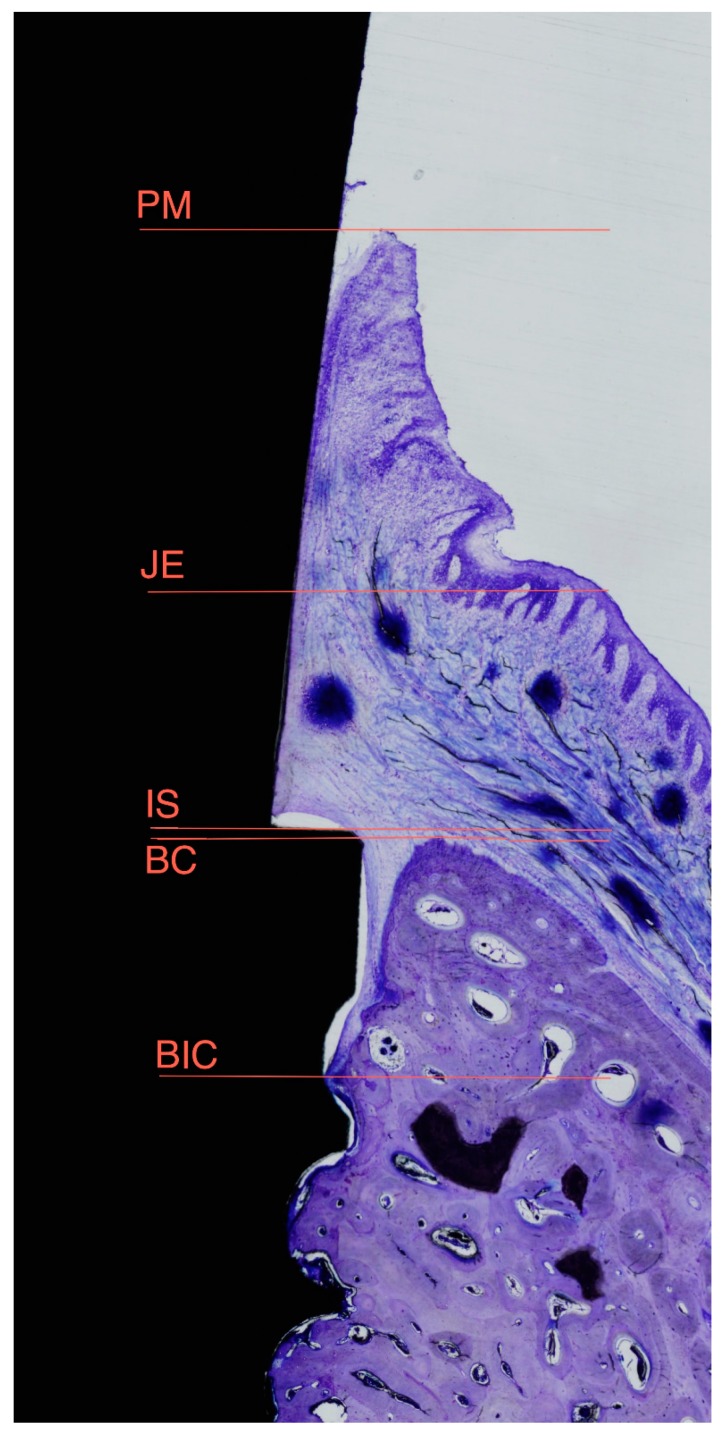
Diagrams representing landmarks used for histometric evaluation: PM, top of the Peri-implant Mucosa; JE, apical portion of the Junctional Epithelium; IS, Implant Shoulder; BC, Bone Crest; and, BIC, first point of Bone-to-Implant Contact.

The most central sagittal section of each implant was analyzed using calibrated digital images at × 10 magnification under a Leica Q500Mc Microscope (Leica Microsystems, Wetzlar, Germany) equipped with a digital video-camera (Sony DXC-151s 2/3-CCd RGB Color Video Camera, Tokyo, Japan) connected to a computer equipped with MIP 4.5 software (MIcroms Image Processing Software, CID, Consulting Image Digital, Barcelona, Spain). The following measurements were taken in millimeters on the buccal and lingual aspects: IS-BIC: distance from the top of the implant shoulder to the first point of bone-to-implant contact; IS-BC: distance from the top of the implant shoulder to the bone crest; PM-BC: distance from the peri-implant mucosa to the bone crest; PM-JE: distance from the peri-implant mucosa to the apical portion of the barrier epithelium; PM-BIC: distance from the peri-implant mucosa to the first point of bone-to-implant contact; JE-BIC: distance from the apical portion of the barrier epithelium to the first point of bone-to-implant contact; and, PM-IS: distance from peri-implant mucosa to the implant shoulder (Figure 4).

### 2.5. Data Analysis

Mean values and standard deviations were calculated for each outcome variable. Differences between implant design groups and between implants of similar neck design (micro-rings as compared with open-thread) were analyzed using SPSS 20.0.0. Software (SPSS Inc., Chicago, IL, USA) applying the Wilcoxon-Mann-Whitney non-parametric test for paired observations. The significance level was set at *p* < 0.05.

## 3. Results

Eight weeks after implant placement, all implants were integrated in mature mineralized bone. No complications arose and no artifacts occurred during histological processing. All implants showed sufficient stability for loading with abutments at the time of placement.

### Histomorphometric Evaluation

Table 1 shows hard tissue remodeling and Table 2 soft tissue adaptation data for each implant neck design, as well as for implants of similar neck design (micro-rings as compared with open-thread).

In pooled data for implants of similar neck design, the open-thread group showed the best results in terms of its capacity to stabilize hard tissue, presenting significant differences at the bone crest level on both lingual and buccal aspects, and at the first point of bone-to-implant contact on the lingual aspect. Differences between groups were also found in soft tissue measurement (PM-JE distance) on both lingual and buccal aspects, with the open-thread group obtaining the best results. 

Pooled data for different implants in the micro-ring group showed significant bone loss on the lingual aspect (IS-BIC distance), and significant differences in PM-BIC and JE-BIC distances. In the double-spiral open-thread and single thread groups, significant differences in bone loss was found on the buccal aspect (IS-BC) and also in the soft tissue (PM-JE distance) level on the buccal aspect. 

## 4. Discussion

This animal study set out to assess the influence of implant neck design on the preservation of bone crest levels and on soft tissue adaption around implants with abutment loading at the time of placement. Sub-crestal implant placement (around 2 mm below the buccal crest) has been observed to reduce crestal bone resorption when compared with crestal placement [30,31,32,33].

Delgado-Ruiz et al. have argued that the thickness, density, and orientation of connective tissue fibers around healing abutments of different geometries influence collagen fiber orientation. For this reason, an abutment with a profile wider than the implant platform favors oblique and perpendicular orientation of collagen fibers and greater connective tissue thickness [34]. In this context, dental implants with expanded platforms placed in the anterior zone of the maxilla and immediately restored with single crowns registered 1.01 mm of crestal bone loss after a 10-year follow-up [35].

As for neck design, the present findings agree with other dog model experiments, showing that different implant neck designs also affect the amount of bone resorption, which may be because the surface affects the distribution of occlusal loads as soon as implants are loaded [36].

Song et al., found that an implant design with open threads reaching the top of the neck underwent less bone loss than other implant designs in which the threads did not reach the top [37], as well as better bone responses than micro-ring designs. Various other clinical studies have also reported different crestal bone loss outcomes in favor of open-thread neck designs [38]. The results of the present study also found that with open-thread implants, the level of the bone around the implant neck region was significantly higher with the double-spiral open-thread design (Neodent^®^ Facility) than the simple spiral open-thread design (Galimplant^®^ IPX). But, these hard tissue remodeling outcomes differ from those observed by Chowdhary et al. [10] and Hudieb et al. [14] who found that the presence of micro-rings—intended to increase the surface area of the implant, this concept being understood globally and not in isolation to a specific brand of implants with a specific surface—promoted bone formation. But, the present results were similar to Jung et al. [17] and Hansson et al. [24] who found—as we would expect—that the increase in implant neck surface produced by prolonging the spiral thread on the body to the top of the neck decreases crestal bone resorption, while producing a smaller bone-to-implant contact distance.

Bone remodeling is also related to the implant connection. This plays an important role in reducing bone loss at the abutment/implant level associated with Morse taper implants and reduced-diameter platform switching abutments, and in reducing the incidence of peri-implantitis [39,40].

Soft tissue adaptation is one of the most important variables determining the long-term success of dental implants. For this reason, one of the objectives of the present study was to evaluate soft tissue variations between implant neck designs. In measurements that were taken on the buccal aspect, it was found that the presence of micro-rings on the neck significantly increased the height of epithelial tissue; this was also the case on the lingual aspect. No previous studies have compared open-thread neck implants with other implants under the same conditions. The open thread design showed less marginal bone loss, probably because it exerts less crestal bone compression. 

## 5. Conclusions

Within the limitations of animal experimentation, it may be concluded that implants with micro-rings on the neck, in spite of offering greater bone-to-implant contact, generate light bone loss than open-thread implants. Moreover, the outcomes that were obtained IPX implants smooth neck design produced less bone loss in the cervical area, following by Facility implants when compared with the other open thread and microthreaded implant designs. Implant thread design can influence on bone remodeling in the cervical area, related to bundle bone preservation.

## Authors Contributions

Conceptualization: R.J.-S., J.L.C.-G.; Data Curation and Resources: J.E.M.-S.d.V.; Formal Analysis: C.P.A.-M.; Funding Acquisition and Research: J.L.C.-G. and C.P.A.-M.: Methodology: R.J.-S. and J.L.C.-G.; Resources and Software: S.A.G.; Writing—original draft: R.J.-S. and J.L.C.-G.; Writing—review & editing: R.J.-S., M.F.D.; Visualization and Methodology: M.F.-D.; Supervision: J.L.C.-G.

## Abbreviations Definition

mmmillimetersIS-BICdistance from the top of the implant shoulder to the first bone to implant contactIS-BCdistance from the top of the implant shoulder to the bone crestPM-BCdistance from the peri-implant mucosa to the bone crestPM-JEdistance from the peri-implant mucosa to the apical portion of the barrier epitheliumPM-BICdistance from the peri-implant mucosa to the first bone to implant contactJE-BICdistance from the apical portion of the barrier epithelium to the first bone to implant contactPM-ISdistance from peri-implant mucosa to the Implant shoulder

## Figures and Tables

**Table 1:** Histomorphometric evaluation of hard tissue remodeling after eight weeks healing. Grouped data are presented for each neck design and for different types of implants with similar designs (micro-rings compared with open-thread). Results are expressed as mean ± standard deviation.

Type of Implants	IS-BC		IS-BIC	
	B	L	B	L
Grouped design Micro-rings	1.61(1.05a	0.89(1.10) a	1.76(0.77)	1.59(0.89) a
Open-Thread	0.55(1.04) a	−0.08(0.67) a	1.03(0.66)	0.63(0.53) a
Micro-rings BlueSky	1.68(0.32)	1.00(0.62)	1.68(0.32)	1.05(0.56) b
Micro-rings C1	1.53(1.55)	0.78(1.52)	1.84(1.11)	2.14(0.85) b
Open-thread IPX	1.45(0.34) c	0.35(0.41)	1.49(0.28)	0.73(0.23)
Open-thread Facility	1.52(0.55) c	0.48(0.44)	1.51(0.53)	0.61(0.63)

(a) *p* < 0.05 for grouped neck designs; (b) *p* < 0.05 for different implants with micro-rings; (c) *p* < 0.05 for different implants with open-thread design.

**Table 2:** Histomorphometric evaluation of soft tissue adaption after 8 weeks healing. Grouped data are presented for each neck design and for implants of similar design (micro-rings compared with open-thread). Results are expressed as mean ± standard deviation.

Type of Implants	PM-BC		PM-BIC		PM-JE		JE-BIC		PM-IS	
	B	L	B	L	B	L	B	L	B	L
Grouped Micro-rings	3.23(0.88)	2.33(0.35)	3.38(0.77)	3.04(0.97)	1.50(0.36) a	1.39(0.40) a	1.88(0.66)	1.65(0.86)	1.62(1.19)	1.44(0.92)
Open-Thread	2.57(0.93)	1.69(0.80)	3.03(0.80)	2.41(0.84)	1.08(0.42) a	0.92(0.46) a	1.94(0.73)	1.49(0.75)	2.00(0.61)	1.78(0.65)
Micro-rings Blue Sky	3.40(0.90)	2.29(0.13)	3.40(0.90)	2.34(0.11) b	1.49(0.20)	1.32(0.16)	1.90(0.89)	1.01(0.25) b	1.71(0.92)	1.29(0.66)
Micro-rings C1	3.06(0.92)	2.38(0.51)	3.37(0.72)	3.73(0.16) b	1.51(0.51)	1.45(0.56)	1.85(0.43)	2.28(0.78) b	1.52(1.53)	1.59(1.20)
Open-thread IPX	3.15(0.68)	1.79(0.79)	3.19(0.63)	2.38(0.68)	1.34(0.20) c	1.00(0.32)	1.85(0.16)	1.37(0.40)	1.69(0.52)	1.44(0.47)
Open-thread Facility	2.20(0.91)	1.68(1.09)	3.00(1.00)	2.55(1.14)	0.86(0.39) c	0.86(0.57)	2.13(0.99)	1.69(1.15)	2.30(0.57)	2.01(0.53)

(a) *p* < 0.05 for grouped neck design; (b) *p* < 0.05 for different implants with micro-rings; (c) *p* < 0.05 for different implants with open-thread design.
